# Higher risk of cam regrowth in adolescents undergoing arthroscopic femoroacetabular impingement correction: a retrospective comparison of 33 adolescent and 74 adults

**DOI:** 10.1080/17453674.2019.1678091

**Published:** 2019-10-15

**Authors:** Tomoya Arashi, Yoichi Murata, Hajime Utsunomiya, Shiho Kanezaki, Hitoshi Suzuki, Akinori Sakai, Soshi Uchida

**Affiliations:** a Wakamatsu Hospital of University of Occupational and Environmental Health, Japan;; b University of Occupational and Environmental Health, Japan

## Abstract

Background and purpose — The current literature does not clarify the predictors of cam regrowth and poor clinical outcome following hip arthroscopic femoroacetabular impingement (FAI) correction surgery. Therefore, we investigated the prevalence and risk factors of cam regrowth following arthroscopic FAI correction surgery in skeletally immature patients compared with skeletally mature patients.

Patients and methods — 33 teenagers (36 hips as 4 underwent bilateral hip arthroscopies, average age 16.7 [SD 1.6] years, 21 boys [22 hips], 12 girls [14 hips]) undergoing arthroscopic FAI correction and 74 adult controls (74 hips, average age 41 [SD 12] years, 42 men [42 hips], 32 women [32 hips]) were retrospectively reviewed. Postoperative radiographs were obtained, and cam regrowth was evaluated. Clinical characteristics, radiographic findings, arthroscopic findings, and procedures between skeletally immature (SI) and mature (SM) patients were compared. Average follow-up period was 28 months in the SI group and 24 months in the SM group.

Results — Preoperatively, 27 of 36 hips were SI, having either a Risser sign grade ≤ 4 of iliac apophysis or open physes of the proximal femur. Cam regrowth was present in 4 of 27 SI hips. The number of cam regrowth cases was significantly higher in SI patients than in SM patients (0/74 hips). 6 patients required revision hip arthroscopic surgeries (4 men: FAI recurrence due to cam regrowth; 2 women: capsulolabral adhesions). At the last follow-up, the mean modified Harris hip score and nonarthritic hip score were significantly improved postoperatively.

Interpretation — 4 of 27 SI hips (95% CI 0.04–0.3) had bone regrowth after cam resection arthroscopically. Our case series showed a non-negligible risk of cam regrowth in SI patients, especially in male patients and patients aged approximately 15 years.

Femoroacetabular impingement (FAI) is today regarded as the most common cause of hip pain in young athletes, resulting from the abutment between the acetabulum and the bump at the femoral head–neck junction (Ganz et al. 2003). Specifically, a cam deformity may be associated with cartilage delamination and labral tear, predisposing to osteoarthritis. Larger alpha angle in cam deformity is the most important predictor of osteoarthritis risk in FAI patients (Agricola et al. 2013a, 2014, Saberi Hosnijeh et al. 2017).

Arthroscopic management of FAI in adolescents offers higher patient-reported outcome scores than in adults (Byrd et al. 2016, Fabricant et al. 2012, Tran et al. 2013). Recent studies have shown that potential risk factors for poor clinical outcomes of hip arthroscopic management for FAI are hip dysplasia, older age, and joint space narrowing at surgery (Fukui et al. 2015, Nepple et al. 2011, Nicholls et al. 2011). Although arthroscopic hip revision surgery for a residual cam deformity yielded substantially improved outcome measures, these were inferior to those after primary arthroscopic FAI correction surgery (Larson et al. 2014).

Therefore we investigated the incidence and risk factors of cam regrowth after arthroscopic correction in FAI patients. We hypothesized that our results would reveal favorable clinical outcomes following hip arthroscopy for adolescent FAI patients and that the risk for cam regrowth in skeletally immature (SI) FAI patients would be higher than that in skeletally mature (SM) FAI patients (Akel et al. 2013, Kuo et al. 2016).

## Patients and methods

**Figure 1.**
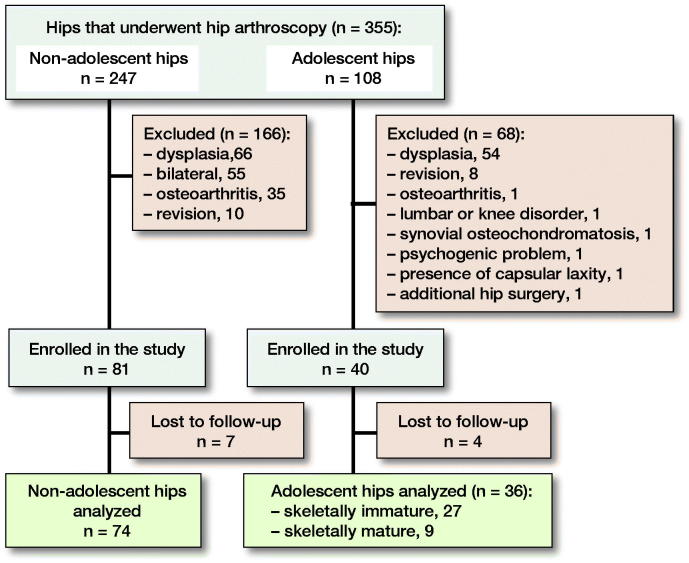
Flowchart showing the recruitment process for patients with femoroacetabular impingement in this study.

355 patients who underwent hip arthroscopies at our institution from January 2009 to December 2014 were enrolled in this study. We defined adolescents as persons aged between 13 and 20 years based on past reports (Fabricant et al. 2012, Sink et al. 2008). Of the remaining 107 potential candidates for this study, ultimately 37 patients (40 hips) were enrolled; 4 patients were lost to follow-up; finally, the outcomes of 33 adolescents (36 hips) who underwent arthroscopy for FAI were investigated (Figure 1).

**Figure 2.**
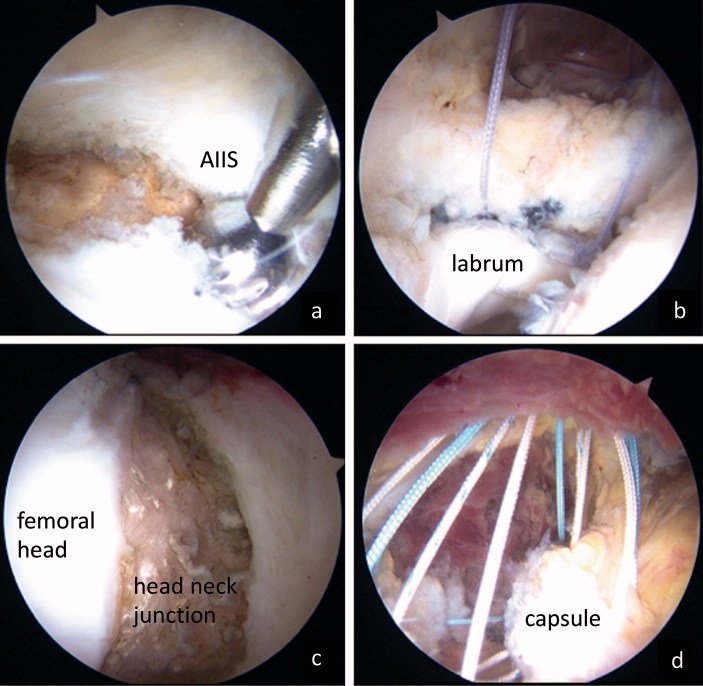
Surgical findings: (a) s motorized burr was utilized to decompress the AIIS and trim the rim; (b) The detached labrum was fixed with suture anchors; (c) cam osteochondroplasty was performed; and the procedure was completed with (d) complete capsular closure using Ultra-braids.

The Risser ossification scale for skeletal maturity was used to evaluate maturity of the pelvis (Bitan et al. 2005). Patients with either a Risser sign of grade ≤ 4 or an open physis of the proximal femur were diagnosed with skeletal immaturity. Patient demographics, radiographs, operative details, validated preoperative, and postoperative modified Harris hip score (MHHS) and nonarthritic hip score (NHS) were collected retrospectively. To compare the clinical outcomes of adolescents and adults, 74 adult patients were recruited from the same cohort during the same time period. Those who were diagnosed with dysplasia or osteoarthritis were excluded.

The indication for arthroscopic FAI correction surgery was based on the physical examination and radiographs of symptomatic patients. The clinical inclusion criteria were refractory groin pain after a minimum of 3 months of nonoperative treatment, including activity modification, physical therapy, and nonsteroidal anti-inflammatory agents; restricted hip range of motion (ROM) (flexion < 105° and/or restricted internal rotation in flexion < 20°); and a positive impingement test. All patients underwent diagnostic intra-articular local anesthesia, which resulted in immediate relief from symptoms in all patients. This effect was temporary (Kalberer 2008, Yamasaki et al. 2015); therefore, we also performed the anterior impingement test and flexion–adduction–internal-rotation test before surgery (Shanmugaraj et al. 2018, Troelsen et al. 2009). Radiographic evidence of a cam deformity included alpha angle > 55° or head–neck offset ratio < 0.14 in at least radiographic view or the presence of a cam lesion on CT or MRI (Clohisy et al. 2008). The alpha angle was measured on plain radiographs. We used the highest alpha angle of the 2 views including modified Dunn view and cross-table lateral view for each hip (Notzli et al. 2002). The radiographic FAI subtype was additionally classified as an isolated cam, an isolated pincer, or a combined FAI. Intra-articular pathological abnormalities, including acetabular labral and chondral lesions, were evaluated by gadolinium-enhanced 1.5 Tesla MR arthrography or 3 Tesla MRI.

We determined the inter-observer and intra-observer reproducibility of these radiographic parameters. For intra-observer reliability, a single hip surgeon measured each radiograph 3 times, with a minimum interval of 1 week between measurements. For inter-observer reliability, the radiographs were independently reviewed and measured by 2 hip surgeons, who were blinded to the clinical data and details of radiology reports. Intraclass correlation coefficients (ICCs) and corresponding 95% confidence intervals (CI) were calculated to quantify inter-observer and intra-observer reliability for continuous variables. The weighted κ-value was used to determine a broken Shenton line and Tonnis classification. κ-values and ICCs of 1.0 were indicative of perfect agreement, and the strength of agreement was interpreted as the following ICC values: 0.80 almost perfect; 0.61–0.80 substantial; 0.41–0.60 moderate; and 0.21–0.40 fair. Based on the standards for the κ-statistic proposed by Landis and Koch, our measurements were in substantial agreement (Landis and Koch 1977).

### Surgical technique (Figure 2)

Supine hip arthroscopy was performed on a traction table with a well-padded perineal post under general anesthesia. Intra-articular pathological abnormalities, including labral tearing and cartilage damage, were assessed by introducing 3 portals: an anterolateral portal, a mid-anterior portal (MAP), and a proximal mid-anterior portal (PMAP). An inter-portal capsular cut was performed to improve the access of the scope and surgical instruments. Then, anteroinferior iliac spine (AIIS) decompression and rim trimming were performed using a motorized round burr to prevent damage to the acetabular labrum if necessary (a), to create a surface for labral healing. The detached labrum was repaired with suture anchors (Gryphon BR, Johnson & Johnson, Raynam, MA, USA) (b). After releasing the traction, the peripheral compartment was evaluated for the presence of a cam lesion. If cam impingement was significant, femoral osteochondroplasty was performed with a 5.5 mm motorized round bur with dynamic confirmation of impingement-free ROM (c). We confirmed that femoral osteoplasty for cam lesion was performed appropriately during surgery under fluoroscopic guidance. Finally, capsular closure through the MAP and PMAP was performed as previously described (d).

### Postoperative recovery

Patients were instructed to avoid full weight-bearing to preserve the repaired labrum and capsule for the first 2 weeks. In cases of a microfracture during surgery, weight-bearing limitations were extended to 6 weeks. Gentle passive ROM exercises such as circumduction were initiated during the 1st week under the supervision of a physical therapist. Continuous passive motion exercises were used to avoid adhesive capsulitis by positioning the hip in 0° to 90° flexion for up to 4 hours a day for 2 weeks.

Endurance strengthening was commenced only after the achievement of maximum ROM, good gait stability, and movement. Patients were allowed to progress to physical activity only after demonstrating symmetric passive ROM, achieving a normal gait pattern, and reporting complete pain resolution.

### Arthroscopic findings

At the time of surgery, the condition of the acetabular rim cartilage was evaluated and classified using the Multicenter Arthroscopy Hip Outcome Research Network (MAHORN) classification (Safran and Hariri 2010). The labrum and ligamentum teres were assessed for tears, and the presence of femoral head chondral lesions was reported using the International Cartilage Research Society (ICRS) classification system (Outerbridge 1961).

### Patient-reported outcome (PRO) scores

Patients completed a comprehensive subjective questionnaire, including the NHS (out of 100 points) (Christensen et al. 2003) and MHHS (out of 100 points) (Byrd 2011), which assessed “pain” and “function,” respectively, to document outcomes.

### Postoperative radiographs

Postoperative radiographs from several viewpoints were taken for each patient at 6 months, 1 year, and 2 years. AP pelvic and false-profile radiographs were obtained as part of the protocol to monitor for osteoarthritis progression, heterotopic ossification, and cam or pincer lesion development. AP pelvic radiographs with the collinear alignment of the symphysis and coccyx were also obtained. In addition, the Dunn or modified Dunn view, cross-table lateral views, frog-leg lateral views, and false-profile views of the hip, before surgery and at annual follow-up, were obtained.

### Cam regrowth

**Figure 3.**
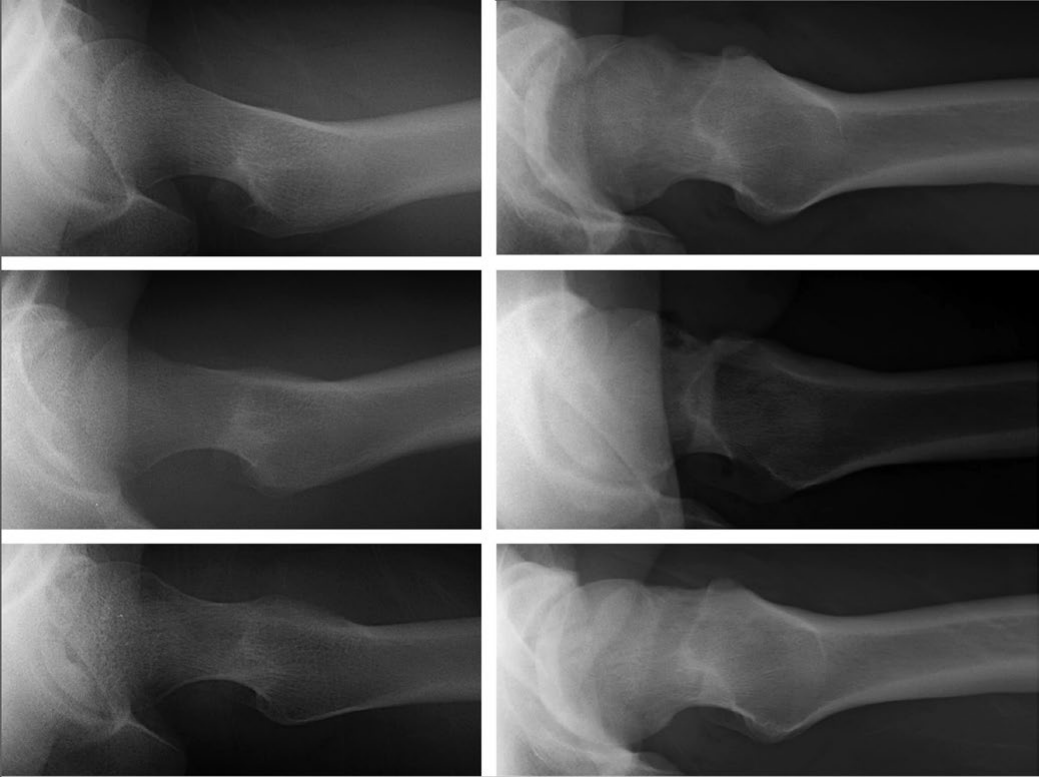
Representative radiographs of cam regrowth.

Cam regrowth was evaluated based on postoperative plain radiographs with the cross-table lateral view and modified Dunn view. To evaluate the alpha angle precisely, we utilized the same view of radiographs and postoperative radiographs. An alpha angle bigger than that measured by radiographs just after surgery was defined as cam regrowth (Figure 3). Patients were evaluated immediately postoperatively, and postoperatively at 6 months, 1 year, and 2 years. The decision to perform revision arthroscopy was based on the patient’s symptom, image evaluation findings including cam regrowth, residual AIIS impingement, and labral re-tear, and physical examination findings. If cam regrowth was found on radiographs images, we performed secondary cam osteoplasty for revision arthroscopy.

### Statistics

Outcome data were analyzed using the t-test, paired t-test, or Fisher’s exact test, comparing the adult and adolescent groups. 95% CI and IQR were calculated. Statistical analyses were performed using the SPSS software package version 21 (IBM Corp, Armonk, NY, USA). Power analysis was performed using the method of Degen et al. (2017). Assuming that postoperative outcomes were compared dependently (paired t-test), the effect size was calculated as d = 1.58, and with the actual statistical power of 0.87, 6 adolescents were required to achieve statistically significant improvement (alpha = 0.05).

### Ethics, funding, and potential conflicts of interest

This study was approved by the institutional review board of University of Occupational and Environmental Health (approval no: H29-005), and by the local institutional review board with a blinded reviewer (approval no. H28-223).

SU is a paid consultant for Smith & Nephew and Zimmer-Biomet and receives research or institutional support from Smith & Nephew/Pfizer.

## Results

### Comparison between adolescents and adults

The cam regrowth rate and revision surgery rate were significantly higher in the adolescent patients than in the adult control group. All other data were similar (Table 1).

**Table 1.** Comparison between adolescent patients and adult controls. Data are presented as mean (SD) [95% CI] or number and percentage (%) [95% CI]

Factor	Adolescent (n = 36)		Adult (n = 74)	
Age at surgery	16.7 (1.7)	[16.1–17.3]	41 (12)	[38–43]
Male sex	22 (61)	[40%–80%]	42 (57)	[45%–68%]
Body mass index	21 (3.4)	[20–23]	22 (2.6)	[22–23]
Lateral center-edge angle (°)	32 (5.4)	[30–34]	35 (6.4)	[33–36]
Alpha angle (°)	63 (13.6)	[58–67]	63 (12.2)	[60–66]
Femoral neck-shaft angle (°)	131 (3.7)	[130–132]	131 (3.9)	[131–132]
AIIS type 1 **^a^**	13 (36)	[20%–52%]	34 (46)	[34%–58%]
Acetabular cartilage delamination, MAHORN III–V	7 (19)	[8.0%–37%]	20 (27)	[17%–37%]
Femoral head cartilage damage, ICRS grade 4	2 (6)	[0%–13%]	9 (12)	[4.5%–20%]
Ligamentum teres pathology	3 (8)	[0%–18%]	11 (15)	[6.6%–23%]
Final follow-up, MHHS	98 (4.6)	[96–99]	97 (4.5)	[96–98]
Final follow-up, NHS	97 (5.7)	[95–99]	93 (11)	[91–96]
Cam regrowth	4 (11)	[0.91%–29%]	0 (0)	
Revision surgery required	6 (17)	[3.9%–29%]	0 (0)	

a AIIS (anteroinferior iliac spine) type was diagnosed using 3-dimensional computed tomography (Hetsroni et al. 2012).

ICRS = International Cartilage Repair Society;

MAHORN = Multicenter Arthroscopy of the Hip Outcome Research Network.

MHHS = modified Harris hip score

NHS = nonarthritic hip score

### Bony maturity and patient demographics

Bony maturity evaluation revealed 27 hips as SI (16 males), having either an open physis of the proximal femur or a Risser grade ≤ 4. For those that were SI at the first operation, 15 patients were SM after 6 months, 4 patients were SM at 1 year, 3 patients were SM after 2 years, 2 patients were SM after 3 years, and 2 patients were still skeletally immature during follow-up, and in 1 patient radiographs were missing. The average follow-up period for SI patients was 24 (SD 13) (IQR 15–32) months, and for SM patients 40 (SD 16) (IQR 36–48) months.

### Arthroscopic findings

No statistically significant differences in the rate of severe acetabular cartilage delamination (MAHORN III–V) was found between the SM group and SI group. Moreover, no significant difference in the rate of femoral head cartilage damage (ICRS grades 2–4) was noted between the 2 groups. Both debridement and labral repair were performed in all cases. Cam osteochondroplasty was performed in all cases in the SI and SM groups.

### Outcomes (PRO scores)

**Figure 4.**
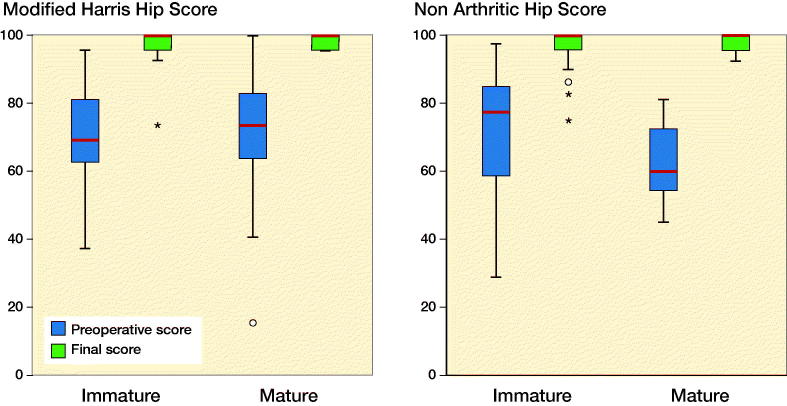
Patient-reported outcome scores: clinical outcome scores of the MHHS and NHS scores are presented with in a box and whisker plot comparing preoperative and final follow-up values; p < 0.001, paired t-test. Red line is median, box is IQR, whiskers are range, and ● and _*_ are outliers.

At follow-up, MHHS and NHS significantly improved in both groups (Figure 4).

### Cam regrowth and revision rate

6 patients subsequently underwent revision hip arthroscopy, including 4 patients who underwent revision surgery because of FAI recurrence due to cam regrowth and 2 because of capsulolabral adhesion. Cam regrowth was noted in 4 hips of 4 patients in the SI group. No cam regrowth occurred in the bilateral cases. The rate of cam regrowth was significantly higher in the SI than in the SM group. All 4 patients with cam regrowth required revision surgery for the recurrence of impingement resulting from cam regrowth. All 4 were boys, and the initial arthroscopy was performed at an average age of 15.6 (range 15.4–15.8) years (Figure 5). In addition, revision hip arthroscopic surgeries were performed in 2 female patients to release adhesions; both were diagnosed with adhesion at the capsulolabral junction and the osteotomy site. Consequently, patients with cam regrowth had a significantly higher rate of revision surgery after initial hip arthroscopy (4 in 6 patients with cam regrowth vs. 0% in patients without cam regrowth).

## Discussion

Our results support the hypothesis that risk factors for poor clinical outcome resulting from cam regrowth include skeletal immaturity and male sex. The main findings of our study were that the rate of cam regrowth, revision surgery rate, and mean NHS at the final follow-up were statistically significantly higher in the adolescent group than in the adult group. 4 SI male patients had significant cam regrowth causing recurrent impingement and required subsequent surgeries. Although 2 female patients required revision surgery because of adhesive capsulitis, female and SM male patients, in general, were more likely to maintain excellent clinical outcomes than SI male patients for a minimum of 2 years postoperatively.

Degen et al. (2017). indicated no evidence of a difference in follow-up survey scores between adult and adolescent groups. Although the improvement in NHS scores was statistically significant at the final follow-up, further long-term clinical follow-up is necessary to ensure that these improved outcome scores are maintained in the adolescent population. Our findings are similar to those of previous studies on hip arthroscopy showing it is a safe procedure, with excellent clinical outcomes in teenagers in the presence of FAI (Philippon et al. 2012). A retrospective case series study of adolescent hips reported that short-term improvement of PRO scores without any complications was possible in selected adolescent athletes (Fabricant et al. 2012). A multicenter case series study of 34 patients (41 hips) under 18 years old showed that hip arthroscopy for treating cam-type impingement resulted in return to sports, significant improvement in PRO scores, and no complications (Tran et al. 2013). Moreover, in a cohort study of 122 hips, superior outcomes were noted in adolescents (≤ 18 years old) to those in the control group (> 18 years old) after arthroscopic management of FAI (Byrd et al. 2016). Kocher et al. (2005) evaluated 54 hips in 42 patients younger than 18 years with a minimum follow-up of 1 year and reported significant improvement in HHS from 53 to 83, with of patients showing improvement. Similarly, our study found that arthroscopic rim trimming, labral repair, and cam osteoplasty provide excellent clinical outcomes for the adolescent FAI.

In our study, we found cam regrowth in 4/27 hips of male adolescents who required revision surgery after hip arthroscopy. Previous studies reported no cam regrowth after hip arthroscopic surgery (Gupta et al. 2014, Perets et al. 2017). The study by Perets et al. (2017) included only female patients. In agreement with the study, our findings showed no cam regrowth in adolescent female patients. Recently, Gupta et al. (2014) demonstrated no cam occurrence after arthroscopic FAI surgery in adolescents. They also compared the mean alpha angle and mean of femoral neck offset at 2 weeks after surgery with those at the final follow-up. In that study, cam regrowth was defined as an alpha angle at the final follow-up bigger than that measured by radiographs just after surgery in each patient. This slight difference in definition may have caused a discrepancy in cam recurrence rate.

Predictors of poor clinical outcomes such as joint-space narrowing on radiographs, a prominent AIIS, residual cam impingement, and capsular laxity after a capsular cut following hip arthroscopy (Nepple et al. 2011, Nicholls et all. 2011), and developmental dysplasia of the hip (Domb et al. 2014, Larson et al. 2014, Fukui et al. 2015, Sardana et al. 2015) have been reported in previous studies. Cam impingement has been reported as a modifiable risk factor and it has also been stated that the early recognition and treatment of this condition prevents arthritic progression in this population (Agricola et al. 2013b). We found no recurrence of cam lesion in the adult control group, whereas cam regrowth was seen in 4/27 hips of adolescent patients. Further, adolescent male FAI patients had a higher rate of cam regrowth requiring revision arthroscopy. Perhaps the fact that these patients underwent initial hip arthroscopy and cam osteochondroplasty at age 15 is crucial. We believe that male sex and age of 15 years at the time of surgery are risk factors for cam regrowth because adolescent male patients around this age have more active bone formation than adults or female patients of similar age. Moreover, the possibility of cam regrowth with mechanical stress on the unfused proximal femoral physis should be considered. These preoperative risk factors should be considered during surgical planning and discussions with male adolescent patients, who are candidates for arthroscopic FAI correction. Agricola et al. (2014) prospectively investigated the development of cam deformity in a series of 63 male pre-professional soccer players. They stated that cam deformities in young male soccer players gradually developed during skeletal maturation and were likely to stabilize upon epiphyseal closure.

Our study has some limitations. First, we lack a nonoperative treatment control group. Since we found no case of cam recurrence in adult patients, the present study focused more on the adolescent population. A study with a larger population may be necessary to allow for additional statistical analyses. Second, arthroscopic findings included chondral lesions, which may have influenced the results. Third, we used MHHS and NHS as the primary clinical outcome assessment scores. Although MHHS shows a significant ceiling effect and iHot should be considered, it was not available during this period (Griffin et al. 2012). Fourth, the sample size was relatively small. However, power analysis suggested that the number of patients was sufficient for this study. Further studies are needed to evaluate the longer-term clinical outcomes of surgical procedures and in a larger number of patients. Fifth, we evaluated cam regrowth on plain radiographs, the projections of which, however, may not be consistent. For evaluation under the same condition we considered performing CT, but this was unrealistic because many patients were concerned about radiation. Sixth, there were 3 bilateral cases in the adolescent group, and our statistical method did not explicitly account for the dependency between bilateral hips in the same patient.

In summary, 4 of 27 SI hips had bone regrowth after CAM resection arthroscopically, a non-negligible risk, especially in male patients and those aged approximately 15 years.
